# Downregulation of the Coiled-Coil Domain Containing 80 and Its Perspective Mechanisms in Ovarian Carcinoma: A Comprehensive Study

**DOI:** 10.1155/2021/3752871

**Published:** 2021-11-15

**Authors:** Zi-Qian Liang, Li Gao, Jun-Hong Chen, Wen-Bin Dai, Ya-Si Su, Gang Chen

**Affiliations:** ^1^Department of Pathology, The First Affiliated Hospital of Guangxi Medical University, No. 6. Shuangyong Rd, Nanning, Guangxi Zhuang Autonomous Region 530021, China; ^2^Department of Pathology, Maternal and Child Health Hospital of Guangxi Zhuang Autonomous Region, No. 59. Xiangzhu Rd, Nanning, Guangxi Zhuang Autonomous Region 530003, China; ^3^Department of Pathology, Liuzhou People's Hospital, NO.8, Wenchang Road, Chengzhong District, Liuzhou, Guangxi Zhuang Autonomous Region 545006, China

## Abstract

**Introduction:**

We aimed to explore the downregulation of the coiled-coil domain containing 80 (*CCDC80*) and its underlying molecular mechanisms in ovarian carcinoma (OVCA). *Materials/Methods*. Immunohistochemical staining was performed to confirm the expression status of *CCDC80* protein. Combining the data from in-house tissue microarrays and high-throughput datasets, we identified the expression level of *CCDC80* in OVCA. We utilized cell-type identification by estimating relative subsets of RNA transcripts (CIBERSORT) algorithm and single-sample gene set enrichment analysis (ssGSEA) to explore the relationship between *CCDC80* and the tumor microenvironment (TME) landscape in OVCA. Pathway enrichment, function annotation, and transcription factor (TFs) exploration were conducted to study the latent molecular mechanisms. Moreover, the cell line data in the Genomics of Drug Sensitivity in Cancer (GDSC) database was used to discover the relationship between *CCDC80* and drug sensitivity.

**Results:**

An integrated standard mean difference (SMD) of −0.919 (95% CI: −1.515–0.324, *P* = 0.002) identified the downregulation of *CCDC80* in OVCA based on 1048 samples, and the sROC (AUC = 0.76) showed a moderate discriminatory ability of *CCDC80* in OVCA. The fraction of infiltrating naive B cells showed significant differences between the high- and low-CCDC80 expression groups. Also, *CCDC80*-related genes are enriched in the Ras signaling pathway and metabolic of lipid. Nuclear receptor subfamily three group C member 1 (*NR3C1*) may be an upstream TF of *CCDC80*, and *CCDC80* may be related to the sensitivity of mitocycin C and nilotinib.

**Conclusion:**

CCDC80 was downregulated in OVCA and may play a role as a tumor suppressor in OVCA.

## 1. Introduction

Ovarian carcinoma (OVCA), a neoplasm in the ovary, originates from embryonic Müllerian ducts and is influenced by hormones and other molecular events [1–6]. As one of the most frequent gynaecological cancers, OVCA is ranked among the deadliest roles from the morbidity and motality perspective [7, 8]. Approximately 21,410 new cases of OVCA have been projected in 2021, which might cause 13,779 deaths in the United States [9]. Patients with OVCA still have poor prognoses despite some treatment approaches, such as chemotherapy, surgery, and immunotherapy [8, 10–12]. Thus, exploring new biomarkers and therapeutic targets for OVCA is imperative.

Coiled-coil domain containing 80 (*CCDC80*), also known as *DRO1* or *SSG1*, is located at 3q13.2. The protein encoded by *CCDC80* is expressed in different cells, such as hepatocytes and adipocytes [13]. Previous studies have identified that *CCDC80* may act as an inhibitor in tumorigenesis of thyroid, pancreatic, and colon cancer [14, 15]. Recently, one study found that low-CCDC80 expression may facilitate the migration of melanoma cells by mediating the downregulation of E-cadherin [16]. Besides, *CCDC80* was proven to be an AIB1-target tumor inhibitor and may participate in the apoptosis of tumor cells [17].

One study had reported that the expression of *CCDC80* mRNA in OVCA tissues was lower than that in nontumor tissues via RT-qPCR [18]. However, no study has revealed the dysregulation of CCDC80 protein in OVCA. Thus, a multicenter study needed to carry out for comprehensively exploring CCDC80 in OVCA. Herein, based on in-house tissue microarrays, RNA-sequencing (RNA-seq), and gene chips, we performed an integrated study and revealed that *CCDC80* was downregulated in OVCA at both the mRNA and protein levels with a large sample size (*n* of OVCA = 802, *n* of non − OVCA = 246). Cell-type identification by estimating relative subsets of RNA transcripts (CIBERSORT) and single-sample gene set enrichment analysis (ssGSEA) was used to explore the relationship between *CCDC80* expression and the tumor microenvironment (TME) landscape of OVCA. Based on Gene Ontology (GO), Kyoto Encyclopedia of Genes and Genomes (KEGG), Disease Ontology (DO), and Reactome enrichment analysis and prediction of transcription factors regulating *CCDC80*, the prospective molecular mechanisms of *CCDC80* in OVCA were explored. Moreover, using cell line data in the Genomics of Drug Sensitivity in Cancer (GDSC) database, we explored the relationship between drug sensitivity on cell lines of OVCA and *CCDC80* expression. All of these works will deepen our understanding of the significance of *CCDC80* in OVCA and explore a latent biomarker and therapy target for OVCA.

## 2. Materials and Methods

### 2.1. Evaluation of CCDC80 Protein Expression in OVCA Tissues

Twenty-four cases of OVCA tissues and 28 cases of non-OVCA controls were collected from the First Affiliated Hospital of Guangxi Medical University, Naning, Guangxi Zhuang Autonomous Region, China. This study was approved by the ethics committee of the First Affiliated Hospital of Guangxi Medical University (no. 2020-KY-E-095). Two tissue microarrays (OVC1021 and OVC2281) were afforded by Pantomics, Inc. (Richmond, CA 94806). Afterward, immunohistochemical (IHC) staining conducted using CCDC80 polyclonal antibody (biorbyt, orb216089, rabbit-anti-human) with 150 OVCA tissues and 46 non-OVCA tissues from clinical samples and tissue microarrays. All operations were performed in accordance with the manufacturer's instructions. Formalin-fixed and paraffin-embedded tissue slides were used to deparaffinize and rehydrate. Then, antigen retrieval was accomplished in a preheated ethylenediaminetetraacetic acid buffer (pH = 9.0). Inactivation of endogenous peroxidase was carried out via 3% H2O2 at room temperature (25°C, the same below) for 15 minutes, and distilled water was used to rinse, followed by PBS soak. The rabbit anti-human CCDC80 polyclonal antibody (dilution 1 : 250) was incubated at 37°C for 90 minutes, followed by PBS rinsing. Universal mouse/rabbit secondary antibody was added into the tissue slides and placed in room temperature for 20 minutes, followed by PBS soak. Coloration was accomplished with diaminobenzidine for 5 minutes, and counterstaining was performed with hematoxylin. Dehydration was carried out in 75%, 85%, 95%, and 100% alcohol successively, and tissue slides were sealed with neutral gum finally. The assessment was conducted via microscope. Blue represented negative staining and red represented positive staining.

Two pathologists evaluated the results of IHC independently. The score of staining intensity followed the criteria: no staining (point = 0), light staining (point = 1), moderate staining (point = 2), and strong staining (point = 3). The score of positive cells in visual field followed the criteria: 0–5% (point = 0), 6–25% (point = 1), 26–50% (point = 2), 51–75% (point = 3), and > 75% (point = 4). The final IHC score equaled the product of intensity and positive cells score [19].

### 2.2. Data Collection from High-Throughput Databases

To identify the expression of *CCDC80* mRNA in OVCA, we searched Gene Expression Omnibus (GEO), Sequence Read Archive (SRA), ArrayExpress, and Oncomine databases to collect gene chips. The search terms were ovarian carcinoma and the mRNA OR gene. Datasets that met the following requirements were collected: (a) the samples were collected from humans, (b) both OVCA and non-OVCA samples were provided and not below three, (c) the expression and annotation profiles were available, and (d) the expression of *CCDC80* was contained. For those datasets from the same platform, we combined them and used the function “Combat” of the sva package to remove the batch effects. Furthermore, we also explored the Cancer Genome Atlas (TCGA) and the Genotype-Tissue Expression (GTEx) databases and included tertiary RNA-seq data of OVCA and normal ovarian samples, and we calculated log_2_(expression+1) to normalize the data. Figure S1 shows the flow chart. As of May 1, 2021, 14 datasets from eight platforms were included (Table 1). After integrating microarrays, we finally obtained eight high-throughput cohorts for our study: GPL570-OVCA, TCGA_GTEx_ovary, GSE66957, GSE119054, GSE124766, GSE132289, GSE146553, and GSE155310.

### 2.3. Statistical Analysis of CCDC80 Expression in OVCA Tissues

If the data followed the normal distribution, Student's *t*-test was used to compare the expression status of *CCDC80* between OVCA and non-OVCA samples using GraphPad Prism 8 software; otherwise, Wilcoxon test was utilized. We also drew receiver operating characteristic (ROC) curves to evaluate the capacity of *CCDC80* to distinguish OVCA samples from non-OVCA samples. The area under the curve (AUC) > 0.7 was reckoned as having moderate discriminatory capacity. Also, by integrating the in-house IHC data, gene chips, and RNA-seq, the standard mean difference (SMD) was calculated, and a summary of ROC (sROC) curve was drawn using Stata v15.1 software (TX, USA). The chi-squared-based *Q*-test and *I*^2^ statistic were used to assess the heterogeneity. *I*^2^ ≤ 50% and *P* value of *Q*-test ≥ 0.05 mean low heterogeneity, and a fixed effect model should be chosen; otherwise, a random effect model should be used to combine SMD. If the 95% confidence interval (CI) of the SMD does not contain zero, the integrated SMD is statistically significant. Egger's test was used to identify publication bias.

### 2.4. Relationships between *CCDC80* Expression and TME Landscape of OVCA

CIBERSORT, a deconvolution algorithm, can estimate the composition of a cell following a gene expression profile with support vector regression [20]. We explored the relationships between *CCDC80* and tumor-infiltrating immune cells in the TCGA-OVCA cohort via the CIBERSORT algorithm in R v3.6.3 software. Subsequently, ssGSEA was performed to explore the immune-related pathways in OVCA by the GSVA package in R [21]. The file “c7.all.v7.4.symbols.gmt” was downloaded from the Molecular Signature Database (MSigDB, http://software.broadinstitute.org/gsea/msigdb/index.jsp) as the reference immune-related gene set. The limma package of R was used to determine significant immune-related pathways and biological processes between high-and low-CCDC80 expression groups (*P* < 0.05).

### 2.5. Identification of Differentially Expressed and Coexpressed Genes of *CCDC80* in OVCA

First, we calculated Pearson's correlation coefficient *r* of *CCDC80* and other genes in OVCA matrices. The genes with the absolute value of *r* ≥ 0.4 and *P* < 0.05 were recognized as the coexpressed genes (CEGs) of *CCDC80*. The CEGs appearing in at least three matrices were chosen. Simultaneously, we calculated the pooled SMD of each gene in the included OVCA matrices using R software. When the 95% CIs of SMDs lacked 0 and *P* < 0.05, we identified the genes as the differentially expressed genes (DEGs) in OVCA. Subsequently, we overlapped the positive-correlated genes of *CCDC80* with downregulated genes and the negative-correlated genes of *CCDC80* with upregulated genes in OVCA, and the intersection genes were obtained for further research.

### 2.6. GO, KEGG, DO, and Reactome Enrichment Analysis

Intersection genes of CEGs and DEGs were used to conduct GO, KEGG, and DO enrichment analysis via clusterProfiler and the DOSE package of R [22]. The online tool KOBAS 2.0 was used to perform Reactome pathway enrichment [23]. GOplot and enrichplot packages of R were used to visualize the results, and the pathview package was used to draw the pathway graphs. Enriched results with adjusted *P* value (false discovery rate, calculated by Benjamini–Hochberg procedure) < 0.05 were chosen to visualize and for further analysis.

### 2.7. GSEA Based on Broad Institute Cancer Cell Line Encyclopedia Data

We downloaded the RNA-seq data of OVCA cell lines from the Broad Institute Cancer Cell Line Encyclopedia (CCLE) database and divided it into two groups based on high- and low-expression level of *CCDC80*. The file “h.all.v7.4.symbols.gmt” from MSigDB was obtained as a reference gene set.

### 2.8. Exploration of Upstream Transcription Factors of *CCDC80* in OVCA

To explore the molecular regulatory mechanisms of *CCDC80* in OVCA, the Cistrome Data Browser (Cistrome DB) was used to predict the latent transcription factors (TFs) of *CCDC80*. Moreover, we overlapped the predicted TFs, *CCDC80* positive-correlated genes, and downregulated genes in OVCA to screen initial TFs. We drew the seqlogo of the motifs via the ggseqlogo package of R. We used the JASPAR database and FIMO tool in the MEME suite to explore the combining site between motifs and the upstream TSS of *CCDC80* [24, 25]. Concurrently, the chromatin immunoprecipitation sequencing (ChIP-seq) data in Cistrome DB was used to validate whether there were peaks before the TSS of *CCDC80* with the IGV tool.

### 2.9. Relationship between *CCDC80* Expression and Drug Sensitivity in OVCA Cell Lines

We downloaded the RNA-seq data of cell lines and estimated half maximal inhibitory concentration (IC50) of all the screened compounds from GDSC database. Mann–Whitney *U*-test was used to compare the estimated IC50 between high- and low-CCDC80 expression groups of OVCA cell lines using GraphPad Prism 8 software. A two-tailed *P* < 0.05 indicates a statistically significant difference. Compounds with higher IC50 signified that the cell lines of OVCA were not susceptible to the compounds.

## 3. Results

### 3.1. The Expression Status of *CCDC80* in OVCA Tissues

**Figure 1:**
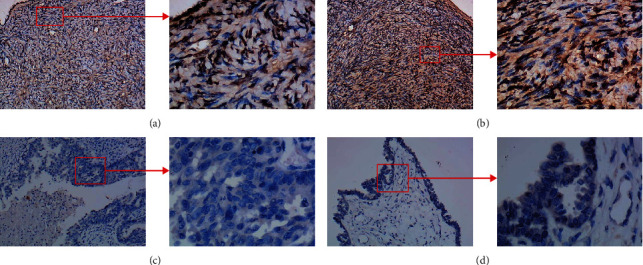
The expression of CCDC80 protein in normal ovary (a) and (b) and ovarian carcinoma (c) and (d) tissues through immunohistochemical (IHC) staining.

**Figure 2:**
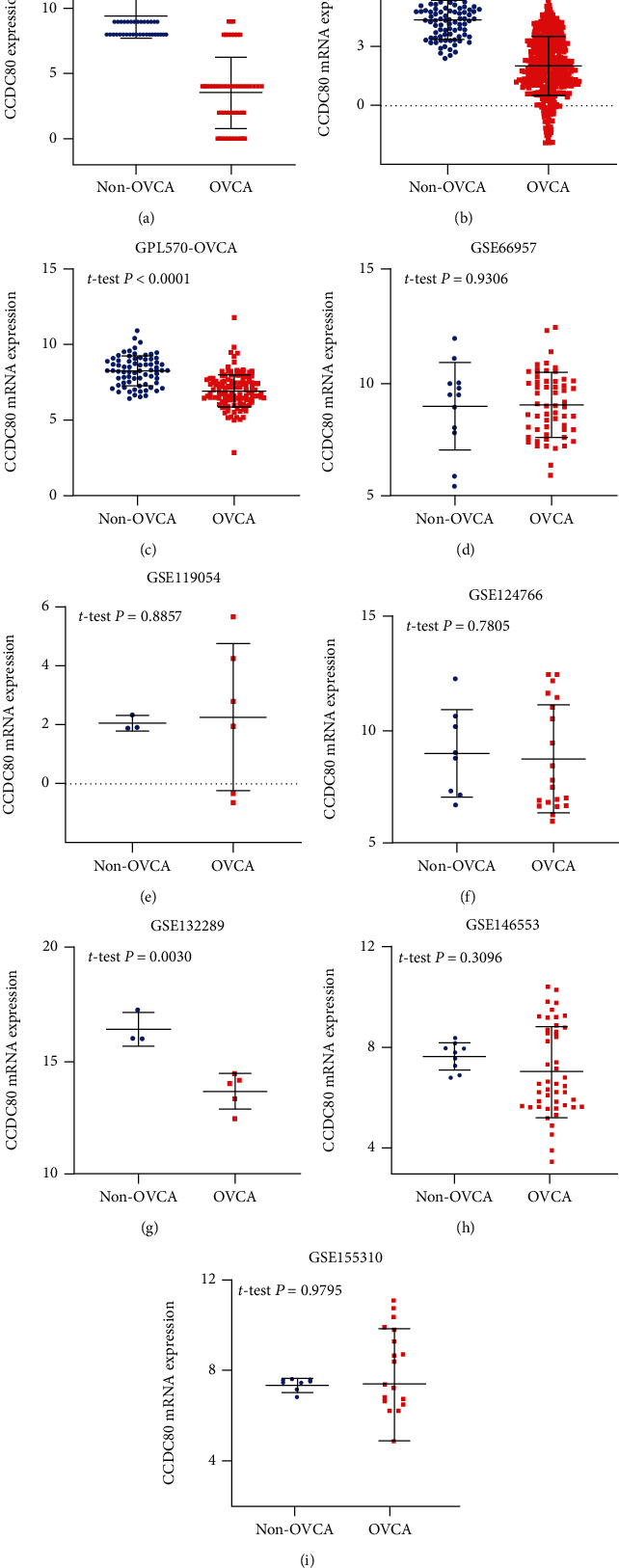
Scatter plots of CCDC80 protein (a) and mRNA (b)–(i) expression of OVCA and the corresponding normal controls.

**Figure 3:**
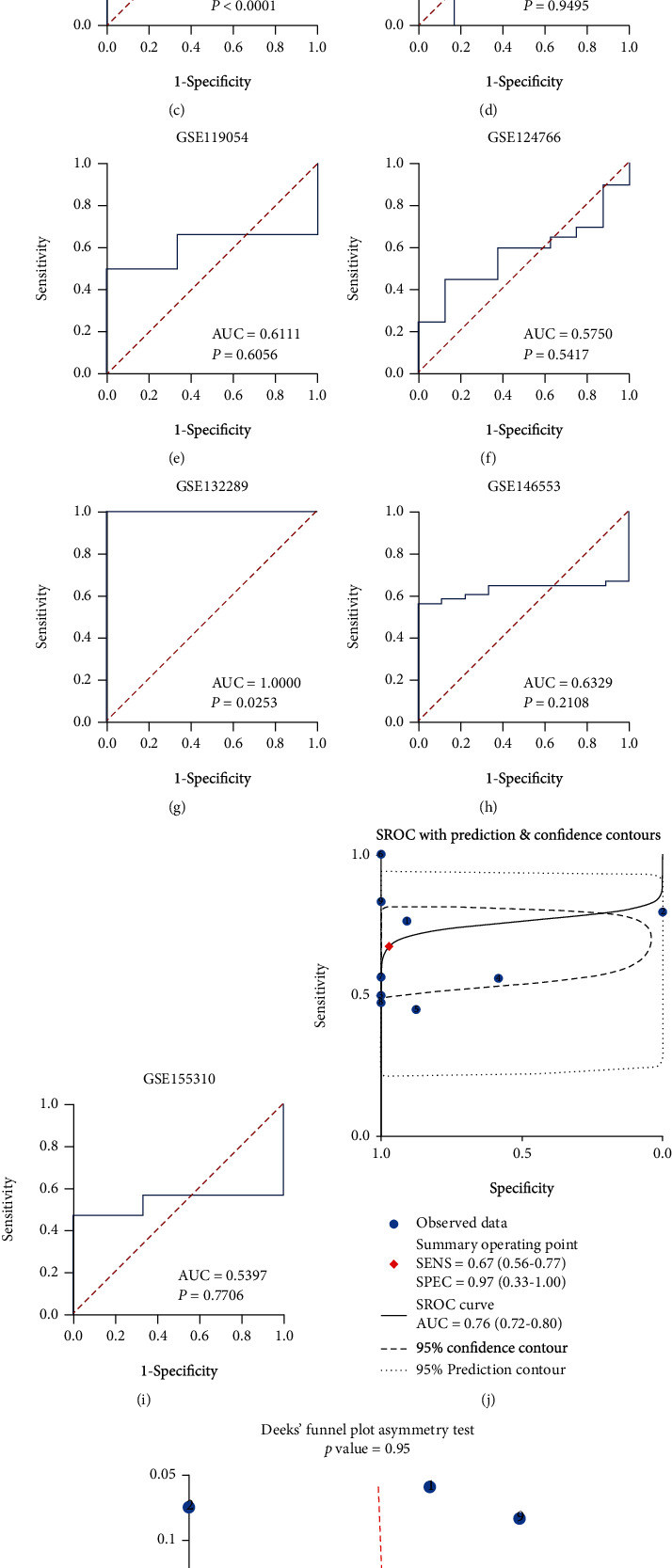
The receiver operating characteristic (ROC, (a)–(i)) and sROC (j) curves of *CCDC80* in OVCA and Deek's test for publication bias test (k).

The results of IHC staining identified that the expression of CCDC80 protein in OVCA tissues was lower than that in non-OVCA tissues (Figure 1), and the difference was statistically significant (*P* < 0.0001, Figure 2(a)). At the mRNA level, three cohorts showed consistent trends with the CCDC80 protein (TCGA_GTEx_ovary, *P* < 0.0001; GPL570-OVCA, *P* < 0.0001; GSE132289, *P* = 0.0030; Figures 2(b), 2(c) and 2(g)). However, the other five datasets showed nonsignificant differences (GSE66957, GSE119054, GSE124766, GSE146553, and GSE155310, *P* > 0.05; Figures 2(d)–2(f), 2(h) and 2(i)). Figures 3(a)–3(i) show the ROC curves.

### 3.2. Comprehensive Evaluation of *CCDC80* in OVCA

**Figure 4:**
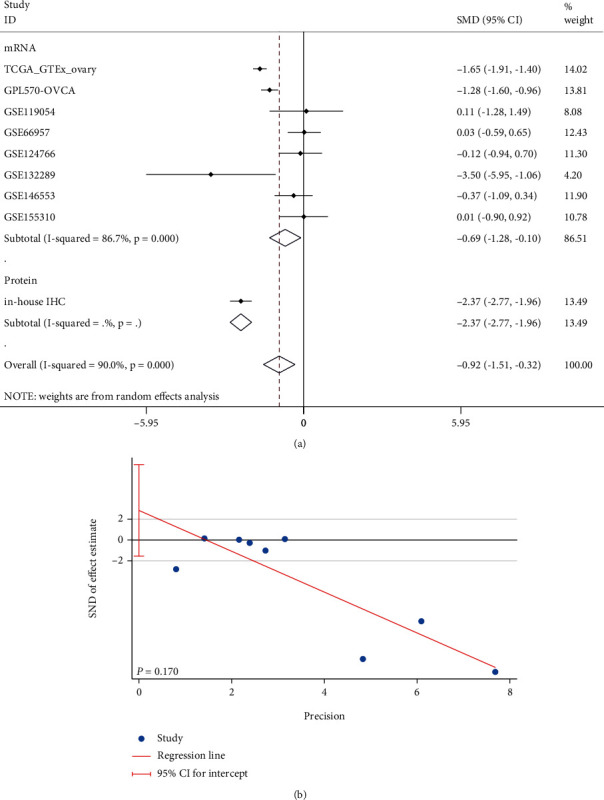
Combined standard mean difference (SMD, (a)) of *CCDC80* expression between OVCA and non-OVCA group and Egger's test (b) for publication bias test (in mRNA group, TCGA_GTEx_ovary was a cohort of RNA-seq, and other cohorts began with “GSE” were gene chip cohorts).

Due to high heterogeneity (*I*^2^ = 90.0%, *P* < 0.001), we used a random effect model to combine SMD. The results of the subgroup analysis showed that *CCDC80* expression in OVCA was below that in the non-OVCA samples at both the mRNA level (subtotal SMD = −0.693, 95% CI: −1.284–−0.101, *P* = 0.022) and the protein level (subtotal SMD = −2.368, 95% CI: −2.774–−1.963, *P* < 0.001). An overall SMD = −0.919 confirmed the downregulation of *CCDC80* in OVCA (95% CI: −1.515–0.324, *P* = 0.002, Figure 4(a)). Egger's test identified no publication bias (*P* = 0.170, Figure 4(b)). The AUC of the sROC curve was 0.76 (95% CI: 0.72–0.80, Figure 3(j)), and Deek's funnel plot also indicated no publication bias (*P* = 0.949, Figure 3(k)).

Moreover, we downloaded the RNA-seq data from the CCLE database and surprisingly found that *CCDC80* was not expressed in the cell lines of OVCAR5_OVARY, OVCA420_OVARY, OVCA433_OVARY, OC315_OVARY, etc., which made the result of the downregulated *CCDC80* level in OVCA more convincing.

### 3.3. The Relationship between the TME Landscape of OVCA and *CCDC80* Expression

**Figure 5:**
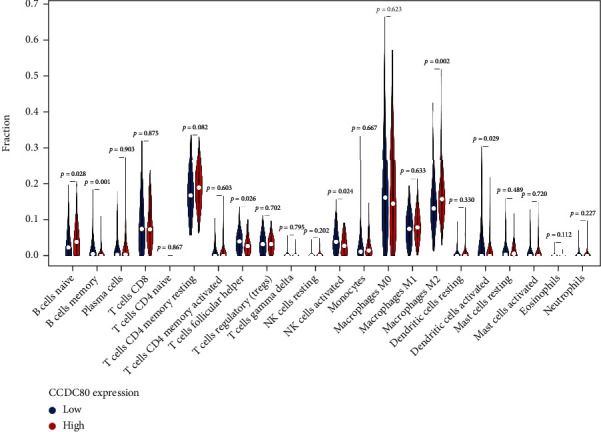
The relationship between *CCDC80* expression and the fraction of immune cell infiltration in OVCA based on CIBERSORT algorithms.

Through CIBERSORT, we found that the fraction of tumor-infiltrating naive B cells and M2 macrophages (M2) was lower in the high-CCDC80 group than in the low-CCDC80 group (naive B cells, *P* = 0.028; M2, *P* = 0.02, Figure 5). However, the fraction of memory B cells (Bm), follicular helper T cell (Tfh), and activated NK cells infiltrated in OVCA was higher in the high-CCDC80 group than in the low-CCDC80 group (Bm, *P* = 0.001; Tfh, *P* = 0.026; activated NK cells, *P* = 0.024; Figure 5).

**Figure 6:**
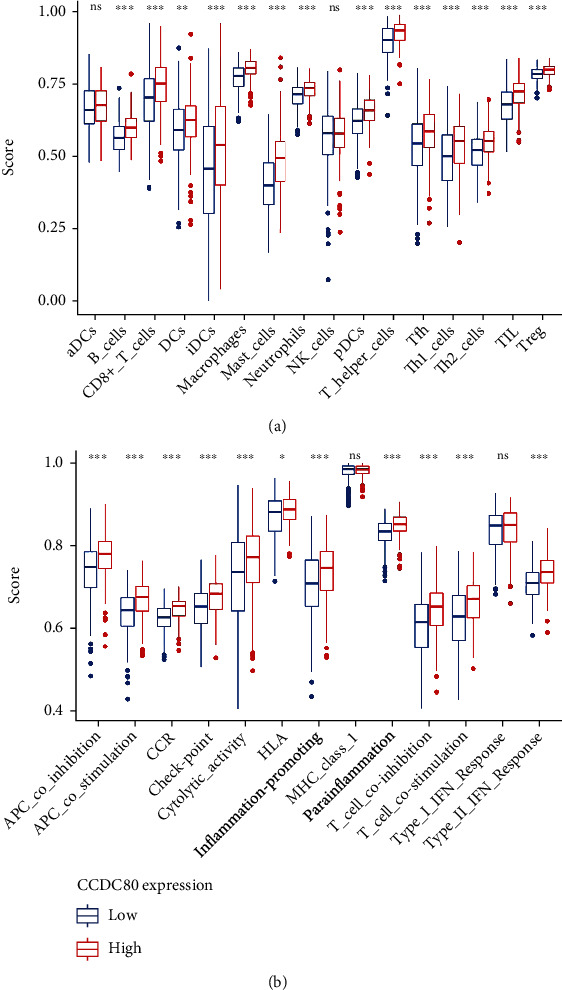
The boxplots visualizing the single-sample gene set enrichment analysis (ssGSEA) in high- and low-*CCDC80* expression group in OVCA based on immunocyte-related gene sets (a) and immune function-related gene sets (b) (^∗∗∗^, *P* < 0.0001; ^∗∗^, *P* < 0.001; ^∗^, *P* < 0.05; ns, *P* ≥ 0.05).

Moreover, the results of ssGSEA showed that between high- and low-*CCDC80* groups, the scores of “B_cells,” “CD8 + _T_cells,” “Th1_cells,” “Th2_cells,” and other immunocyte-related gene sets were statistically significant (Figure 6(a)). Also, the scores of “APC_co_inhibition,” “APC_co_stimulation,” “Check-point,” “Type_II_IFN_Response,” and other immune function-related gene sets were statistically significant between the high- and low-CCDC80 groups (Figure 6(b)). Interestingly, the score in the high-CCDC80 group considerably exceeded that in the low-CCDC80 group.

### 3.4. Enrichment Analysis

Through intersection, we obtained 298 *CCDC80*-related downregulated DEGs and 156 *CCDC80*-related upregulated DEGs (Figure S2). The results of GO term annotation showed that *CCDC80*-related downregulated DEGs were relative to “focal adhesion,” “cell-cell junction,” and “glycosaminoglycan binding,” and that CCDC80-related upregulated genes were enriched in “cell-cell adhesion mediator activity,” “microtuble,” and “cadherin binding involved in cell-cell adhesion” (Figure S3). Regarding DO enrichment, *CCDC80*-related downregulated DEGs may participate in some pulmonary and cardiovascular diseases, while *CCDC80*-related upregulated DEGs may participate in ovarian tumors and urinary system cancer (Figure S4).

**Figure 7:**
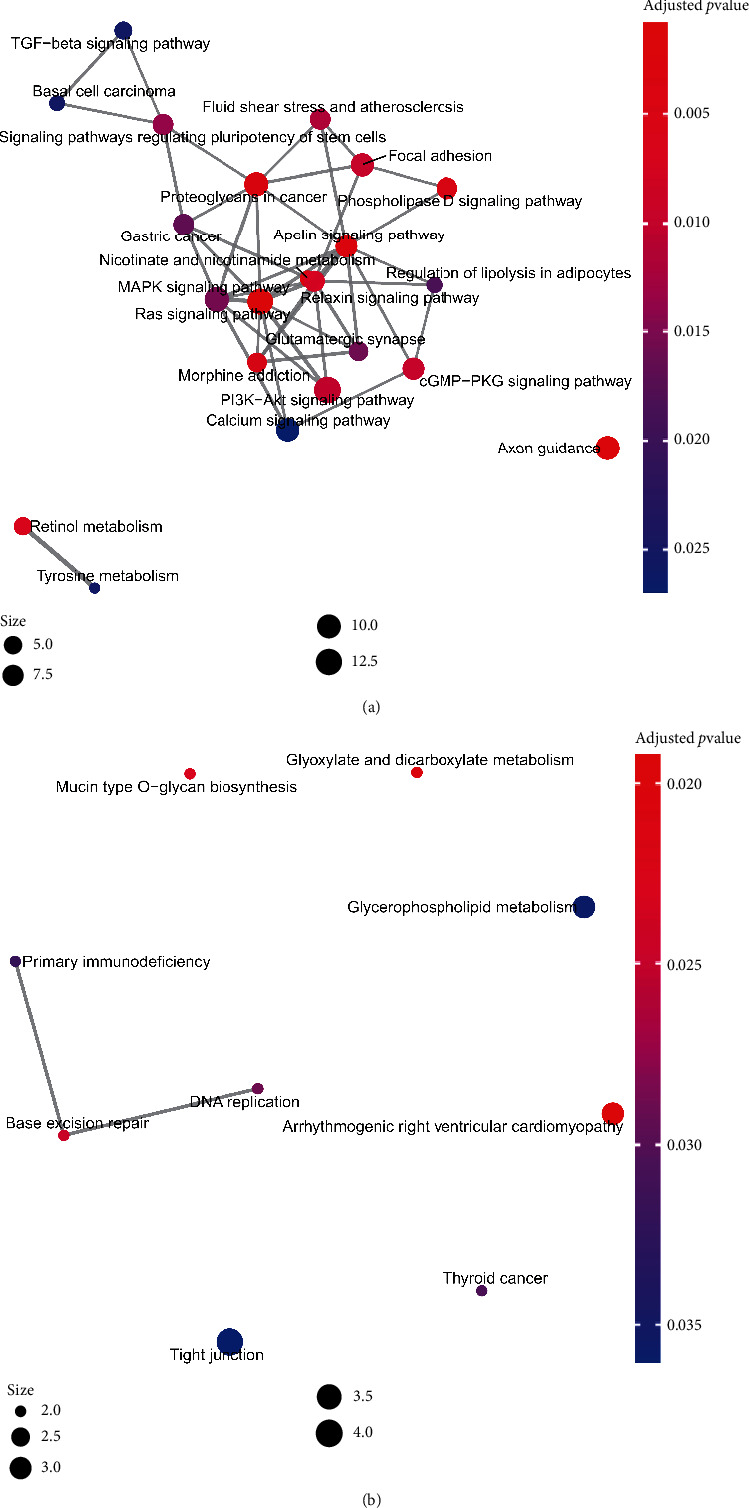
KEGG enrichment plots of the intersection genes from *CCDC80* positively related coexpressed genes (CEGs) and downregulated differentially expressed genes (DEGs) (a), and *CCDC80* negatively related CEGs and upregulated DEGs (b).

Moreover, the results of KEGG enrichment identified that *CCDC80*-related downregulated DEGs were enriched in the “Ras signaling pathway,” “Axon guidance,” and “Proteoglycans in cancer,” etc., while *CCDC80*-related upregulated DEGs may be involved in “DNA replication” and “Base excision repair,” etc. (Figure 7, Table S1). The particulars of the Ras signaling pathway are demonstrated in Figure S5, which illuminates how the Ras signaling pathway may be related to some vital pathways in cancer, such as cell-cell junctions, cell migration, MAPK signaling, and the PI3K-Akt signaling pathway. Similarly, proteoglycans in the cancer pathway were also related to tumor-related pathways, such as cell adhesion, apoptosis, oncogenic signaling, tumor cell migration, and invasion pathway (Figure S6).

Meanwhile, the results of Reactome analysis revealed that *CCDC80*-related downregulated DEGs may be relative to some metabolism-related pathways, such as “Integration of energy metabolism,” “Metabolism of lipids,” “Triglyceride metabolism,” and “Metabolism of vitamins and cofactors,” while *CCDC80*-related upregulated DEGs are enriched in some cell cycle-related pathways, such as “Cell cycle,” “M phase,” and “Cell cycle checkpoint” (Figure S7).

**Figure 8:**
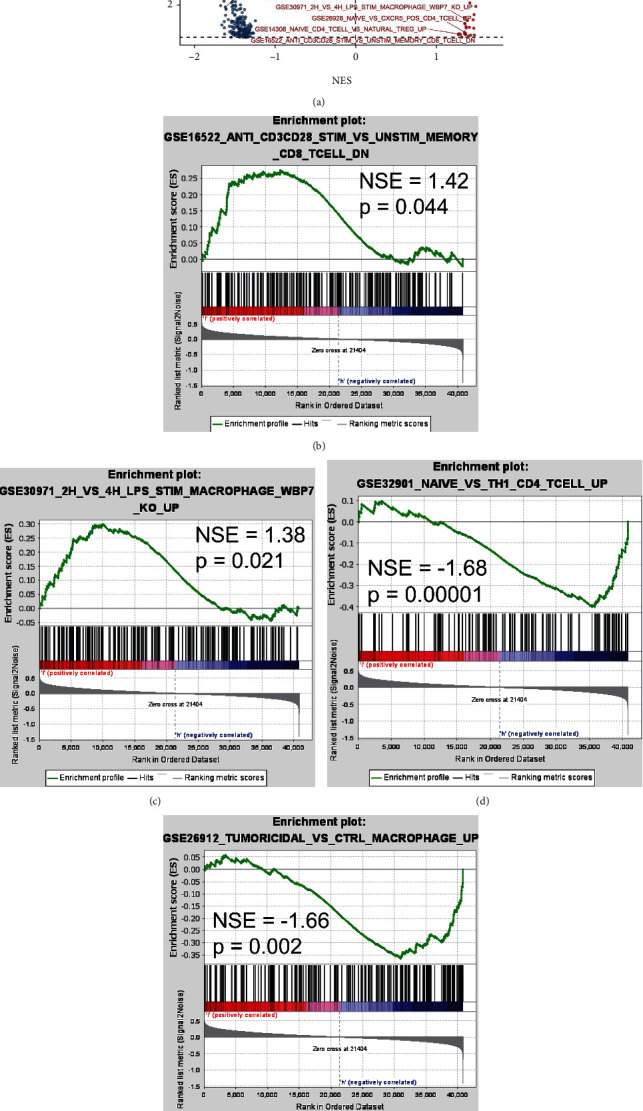
A volcano plot shows the results of GSEA based on the OVCA cell lines and enrichment plots of immune-related gene sets in low-*CCDC80* group (b) and (c) and high-*CCDC80* group (d) and (e).

Following the cell line of OVCA, GSEA revealed that high- and low-CCDC80 groups were both enriched in some immune-related gene sets, such as “GSE26912_TUMORICIDAL_VS_CTRL_MACROPHAGE_UP,” “GSE30971_2H_VS_4H_LPS_STIM_MACROPHAGE_WBP7_KO_UP,” and “GSE32901_NAIVE_VS_TH1_CD4_TCELL_UP” (Figure 8).

### 3.5. The Potential of TF Regulatory *CCDC80* in OVCA

**Figure 9:**
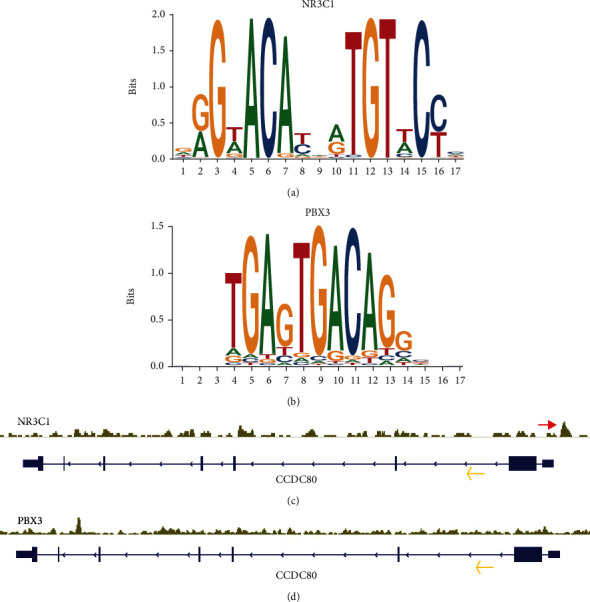
Seqlogos of the motifs of transcription factor *NR3C1* (a) and *PBX3* (b); ChIP-seq peak (see red arrow) of *NR3C1* in the upstream of the transcription start site of *CCDC80* (c); and the yellow arrow indicates the transcription direction of *CCDC80*; no ChIP-seq peak of *PBX3* in the upstream of the transcription start site of *CCDC80* (d).

By overlapping the predicted TFs from Cistrome DB, positive-correlated genes of *CCDC80*, and downregulated genes in OVCA, we obtained two initial TFs (*NR3C1*, *PBX3*) regulating *CCDC80* (Figure S8). The motifs of *NR3C1* and *PBX3* are demonstrated in Figures 9(a) and 9(b), and a ChIP-seq peak of *NR3C1* was observed before TSS of *CCDC80* (Figure 9(c)). However, the ChIP-seq peak of *PBX3* was missing before the TSS of *CCDC80* (Figure 9(d)), which indicated that *PBX3* may not be the regulatory TF of CCDC80. Using the JASPAR and FIMO tools, a perspective binding sequence in common was obtained—AAGAAAAGAATGTAGCC.

### 3.6. The Relationship between *CCDC80* Expression and Drug Sensitivity in OVCA Cell Lines

**Figure 10:**
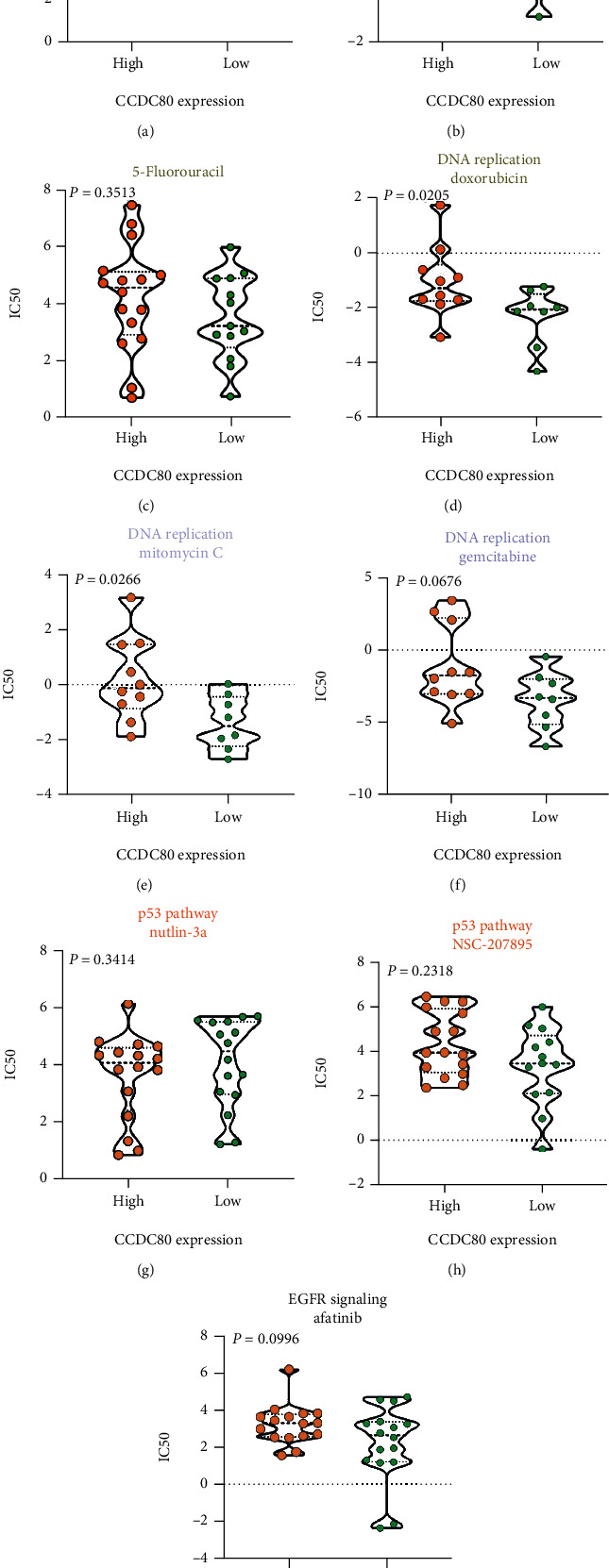
Violin plots visualizing the differences of estimated half maximal inhibitory concentration (IC50) of compounds between high- and low-*CCDC80* expression group.

By comparing the estimated IC50 between high- and low-*CCDC80* cell lines of OVCA, we found that the estimated IC50 of the ABL signaling inhibitor (nilotinib and tipifarnib) and DNA replication inhibitor (doxorubicin and mitomycin C) in the high-CCDC80 group exceeded that in the low-CCDC80 group (Figures 10(a), 10(b), 10(d), and 10(e)). However, the IC50 of some classic anticarcinogens, such as 5-fluorouracil and afatinib, showed a nonsignificant difference between the two groups (Figures 10(c) and 10(i)).

## 4. Discussion

In this study, we revealed the downregulation of CCDC80 at the protein level based on tissue microarrays (number of OVCA = 150, number of non − OVCA = 46). Via RNA-seq and gene chip data, we substantiated this decreasing trend at the mRNA level with a large sample size (number of OVCA = 652, number of non − OVCA = 200) and different approaches (*t*-test and combined SMD). Cocurrently, we identified that the expression of *CCDC80* was related to the TME landscape in OVCA using the CIBERSORT algorithm and ssGSEA. Also, we found that *NR3C1* may be a potential upstream TF of *CCDC80*. Moreover, following the RNA-seq in cell lines and IC50 of compounds, we identified that the expression status of *CCDC80* may have a relationship with drug sensitivity.

In previous study, 21 OVCA samples were used to detect the low expression of *CCDC80* mRNA by RT-qPCR [18]. However, no study reported the expression status of *CCDC80* at both mRNA and protein level in OVCA with multiple detection means and multicenter samples (based on the PubMed database, as of May 16, 2021). Herein, we conducted a subgroup analysis to calculate integrated SMD and first revealed that *CCDC80* expression in OVCA tissues was below that in non-OVCA tissues with 1048 multicenter samples via multiple approaches (IHC, gene chips, and RNA-seq).

The clinical significance of *CCDC80* in malignant tumors was attractive. In previous studies, *CCDC80* was reported as a prognostic signature in serous ovarian carcinoma, colorectal cancer, and muscle-invasive bladder cancer [26–28]. However, no study has revealed the discriminatory capacity of *CCDC80* in malignant tumors. In our study, an AUC = 0.76 (95% CI: 0.72–0.80) of sROC indicated a moderate ability of *CCDC80* to distinguish OVCA from nontumor ovary. Unfortunately, due to the small clinical sample size and lack of follow-up information, the relationship between *CCDC80* and clinical parameters and the prognostic value of *CCDC80* in OVCA was unexplored.

Despite neoplastic cells, the components of tumors have numerous normal cells incorporating fibroblasts, inflammatory immunocytes, and epithelial cells [8, 29]. Many studies have illustrated that TME may play a dynamic role in the biological behaviors of tumors and may be a potential therapy target of OVCA [8, 30–34]. TME is an essential element to consider when stimulating the antitumor immunoreaction since TME contains many types of immunocytes and stromal cells. For example, tumor-infiltrating CD20^+^ B-cells, such as naive B-cells, were found to act as antigen-presenting cells and to facilitate antitumor immunity and may negatively regulate tumor growth [35–37]. Tumor-infiltrating B-cell can expedite the tumor antigens present to stimulate the function of T lymphocytes via upregulating costimulatory molecules (such as CD80/86) and HLA-II [38]. Existing evidence has shown that some B-cell-related pathways (such as CCL19, 21/CCR7, and CXCL13/CXCR5 axes) can induce the formation of tertiary lymphoid structures and activate the local antitumor immune response [39]. In the present study, through the CIBERSORT algorithm and ssGSEA, we found that the high expression of *CCDC80* was related to a high fraction of infiltrating naive B-cells and a high score of B-cell-related pathways, which revealed that *CCDC80* may act as a tumor suppressor via effecting B lymphocytes. The result of GSEA following the OVCA cell line showed that *CCDC80* may participate in some immune-related biological processes, but these still need further research.

We performed KEGG pathway enrichment analysis and found that positive-related DEGs of *CCDC80* were enriched in Ras signaling and proteoglycans in the cancer pathway. A study has reported that the Ras signaling pathway activates the tumor-related fibroblast and stimulates the proliferation of cancer cell [40]. Another study found that Ras signaling may participate in the process of prostate cancer metastasis to bone via interaction with Wnt signaling [41]. Proteoglycan is a type of biomacromolecule comprising a protein core and glycosaminoglycan. Proteoglycan is an essential regulatory factor of the extracellular matrix (ECM) and can implicate the biological behaviors of cells through interaction with cytokines, adhesion moleculars, or growth factors, which are critical in tumorigenesis and tumor metastasis [42, 43]. Moreover, proteoglycan can affect TME and tumor-related immune responses and even participate in metabolic reprograming [42, 44]. However, the impact of the Ras signaling pathway and proteoglycans on OVCA has been partially explained. Following the KEGG results, we inferred that the downregulated *CCDC80* may impact the Ras signaling pathway and proteoglycan and may be involved in the tumorigenesis and development in OVCA, which still needs more validation.

Regarding the metabolic process and pathways, the metabolism of lipids was significant in our Reactome analysis. Existing evidence has revealed that two lipids (arachidonic acid and lysophosphatidic acid) relate to the dysregulated Ca^2+^ channels and Ca^2+^-activated potassium and impact cell migration and invasion in OVCA [45]. Furthermore, the metabolism of lipids was proved to be considerable for maintaining cancer stem cells, and the level of unsaturated lipids in OVCA stem cells was significantly high, which indicated that the lipid-related metabolic process may be the potential therapeutic target for OVCA [46–48]. A study has reported that *CCDC80* may be an inhibitor in the metabolism of lipids and adipogenesis [49]. In our study, we inferred that the downregulated *CCDC80* may influence the metabolism of lipids and facilitate the development of OVCA.

To further study the underlying molecular mechanisms of downregulated *CCDC80* in OVCA, we explored the upstream regulatory TFs of *CCDC80*. Previously, *CCDC80* was reported as a downstream target gene for TFs YAP/TAZ [50]. In the current study, we identified that *NR3C1* may be a potential TF regulating *CCDC80* in OVCA, which clarifies the molecular mechanisms of *CCDC80* in OVCA.

Nilotinib, a type of tyrosine kinase inhibitor, was used to treat chronic myeloid leukemia [51, 52]. A study reported that nilotinib induces the apoptosis of OVCA cells via a mitochondrion-dependent process [53]. Tipifarnib, a highly selective farnesyltransferase, was reported to induce apoptosis, tumorigenesis cease, and regression of head and neck squamous cell carcinoma *in vivo* [54]. Also, tipifarnib may reduce the viability of OVCA cells *in vitro* [55]. Mitomycin C is a well-known antitumor drug that can form deoxyadenosine monoadducts with DNA and block the replication of DNA to impede the proliferation of cancer cell [56]. One clinical trial found that mitomycin C plus cisplatin has a promising effect in treating recurrent BRCA1-related OVCA [57]. Though these drugs tended to have a potential capacity in the treatment of OVCA, the resistance of drugs was common recently [58–60]. Some studies have reported the mechanisms and prediction biomarkers of resistance [61–63], whereas more exploration needed to carry out concerning the chemotherapeutic resistance. In our study, we found the estimated IC50 of nilotinib, tipifarnib, and mitomycin C in high-*CCDC80* OVCA cells exceeded that in low-*CCDC80* OVCA cells, which indicates *CCDC80* is expected to be a biomarker to forecast the sensitivity of antineoplastic drugs. Our results also identified the dysregulation of CCDC80 in OVCA might play a role in the resistance of chemotherapy. But it still needs experiments and large-scale clinical trials for further verification.

Overall, our study demonstrated the downregulated trend of *CCDC80* at both the mRNA and protein levels in OVCA, and *CCDC80* may act as a tumor suppressor by affecting the TME and metabolism. Nevertheless, there were still some limitations. First, the collection of clinical samples and clinicopathological parameters was limited, making the clinical value of *CCDC80* not to be revealed. Furthermore, the molecular mechanisms of *CCDC80* and drug sensitivity still need further research and validation via experiments *in vitro* and *in vivo* and large-scale clinical trials.

## 5. Conclusion

Briefly, by combining the data from in-house IHC and a high-throughput database, we revealed that *CCDC80* was downregulated in OVCA and that CCDC80 probably has an intimate relationship with TME and metabolism in OVCA. Moreover, we identified that *NR3C1* may be a latent TF regulating *CCDC80* and that CCDC80 may be an indicator to forecast drug sensitivity, but it needs further exploration.

## Figures and Tables

**Table 1:** General characteristics of microarray and RNA-sequencing datasets on ovarian carcinoma.

Study	Test method/platform	Country	Year	OVCA group	Noncancerous ovary controls
GSE105437	GPL570	South Korea	2017	10	5
GSE29450	GPL570	USA	2011	10	10
GSE18520	GPL570	USA	2009	53	10
GSE10971	GPL570	Canada	2008	13	24
GSE54388	GPL570	USA	2017	16	6
GSE14407	GPL570	USA	2009	12	12
GSE36668	GPL570	Norway	2012	4	4
GSE119054	GPL19615	China	2019	6	3
GSE66957	GPL15048	USA	2015	57	12
GSE146553	GPL6244	USA	2020	46	9
GSE124766	GPL6480	Germany	2020	20	8
GSE132289	GPL20301	UK	2020	5	3
GSE155310	GPL18573	UK	2020	21	6
TCGA_GTEx_ovary	RNA-seq	USA	2021	379	88

OVCA: ovarian carcinoma.

## Data Availability

The original data are available from the corresponding author upon reasonable request.

## Supplementary Materials

Supplementary Materials Table S1: Kyoto Encyclopedia of Genes and Genomes analysis of the intersection of *CCDC80* related coexpressed genes and differentially expressed genes. Figure S1: the flow chart of screening OVCA-related datasets for this study. Figure S2: Venn diagrams for the intersection genes consists of CCDC80 positively related CEGs and downregulated DEGs (a), and intersection genes consist of CCDC80 negatively related CEGs and upregulated DEGs (b). Figure S3: bubble plots of GO annotation analysis based on intersection genes from CCDC80 positively related CEGs and downregulated DEGs (a), and CCDC80 negatively related CEGs and upregulated DEGs (b). Figure S4: Circle plots of DO annotation analysis based on intersection genes from CCDC80 positively related CEGs and downregulated DEGs (a), and CCDC80 negatively related CEGs and upregulated DEGs (b). Figure S5: visualization of Ras signaling pathway. Figure S6: visualization of proteoglycans in cancer pathway. Figure S7: circle plots of Reactome pathway analysis based on intersection genes from CCDC80 positively related CEGs and downregulated DEGs (a), and CCDC80 negatively related CEGs and upregulated DEGs (b). Figure S8: Venn diagrams for the intersection genes consist of CCDC80 positively related CEGs, downregulated DEGs, and predicted TFs from Cistrome DB.
