# Supplementary material for: Downregulation of the Coiled-Coil Domain Containing 80 and Its Perspective Mechanisms in Ovarian Carcinoma: A Comprehensive Study
Source: Int J Genomics. 2021 Nov 15;2021:3752871. doi: 10.1155/2021/3752871 (PMC8608537; doi:10.1155/2021/3752871)
Supplement: Supplementary Materials — Table S1: Kyoto Encyclopedia of Genes and Genomes analysis of the intersection of CCDC80 related coexpressed genes and differentially expressed genes. Figure S1: the flow chart of screening OVCA-related datasets for this study. Figure S2: Venn diagrams for the intersection genes consists of CCDC80 positively related CEGs and downregulated DEGs (a), and intersection genes consist of CCDC80 negatively related CEGs and upregulated DEGs (b). Figure S3: bubble plots of GO annotation analysis based on intersection genes from CCDC80 positively related CEGs and downregulated DEGs (a), and CCDC80 negatively related CEGs and upregulated DEGs (b). Figure S4: Circle plots of DO annotation analysis based on intersection genes from CCDC80 positively related CEGs and downregulated DEGs (a), and CCDC80 negatively related CEGs and upregulated DEGs (b). Figure S5: visualization of Ras signaling pathway. Figure S6: visualization of proteoglycans in cancer pathway. Figure S7: circle plots of Reactome pathway analysis based on intersection genes from CCDC80 positively related CEGs and downregulated DEGs (a), and CCDC80 negatively related CEGs and upregulated DEGs (b). Figure S8: Venn diagrams for the intersection genes consist of CCDC80 positively related CEGs, downregulated DEGs, and predicted TFs from Cistrome DB. [file 3752871.f1.pdf]

Table S1. Kyoto Encyclopedia of Genes and Genomes analysis of the intersection of *CCDC80* related co-expressed genes and differentially expressed genes.

Gene	ID	Description	adjusted *p*-value	Count
CCDC80 postively related upregulated DEGs	hsa05412	Arrhythmogenic right ventricular cardiomyopathy	0.01917	3
	hsa00630	Glyoxylate and dicarboxylate metabolism	0.020539	2
	hsa00512	Mucin type O-glycan biosynthesis	0.023199	2
	hsa03410	Base excision repair	0.02458	2
	hsa03030	DNA replication	0.028917	2
	hsa05216	Thyroid cancer	0.030426	2
	hsa05340	Primary immunodeficiency	0.031966	2
	hsa00564	Glycerophospholipid metabolism	0.035759	3
	hsa04530	Tight junction	0.036096	4
CCDC80 negatively related downregulated DEGs	hsa04014	Ras signaling pathway	0.000842	12
	hsa04360	Axon guidance	0.001457	10
	hsa04371	Apelin signaling pathway	0.002979	8
	hsa00760	Nicotinate and nicotinamide metabolism	0.003266	4
	hsa05205	Proteoglycans in cancer	0.003488	10
	hsa04072	Phospholipase D signaling pathway	0.004771	8
	hsa05032	Morphine addiction	0.005488	6
	hsa00830	Retinol metabolism	0.007029	5
	hsa04926	Relaxin signaling pathway	0.007985	7
	hsa04510	Focal adhesion	0.009477	9
	hsa04022	cGMP-PKG signaling pathway	0.009684	8
	hsa04151	PI3K-Akt signaling pathway	0.010137	13

	hsa05418	Fluid shear stress and atherosclerosis	0.011782	7
	hsa04550	Signaling pathways regulating pluripotency of stem cells	0.013617	7
	hsa04010	MAPK signaling pathway	0.015449	11
	hsa04724	Glutamatergic synapse	0.015783	6
	hsa05226	Gastric cancer	0.016743	7
	hsa04923	Regulation of lipolysis in adipocytes	0.018262	4
	hsa05217	Basal cell carcinoma	0.025394	4
	hsa00350	Tyrosine metabolism	0.025596	3
	hsa04350	TGF-beta signaling pathway	0.025714	5
	hsa04020	Calcium signaling pathway	0.027033	9
	hsa00250	Alanine, aspartate and glutamate metabolism	0.027501	3
	hsa04068	FoxO signaling pathway	0.029023	6
	hsa04933	AGE-RAGE signaling pathway in diabetic complications	0.032445	5
	hsa04015	Rap1 signaling pathway	0.033377	8
	hsa04924	Renin secretion	0.033997	4
	hsa04910	Insulin signaling pathway	0.035049	6
	hsa04080	Neuroactive ligand-receptor interaction	0.040131	11
	hsa04931	Insulin resistance	0.042996	5
	hsa02010	ABC transporters	0.045362	3

DEGs: differentially expressed genes

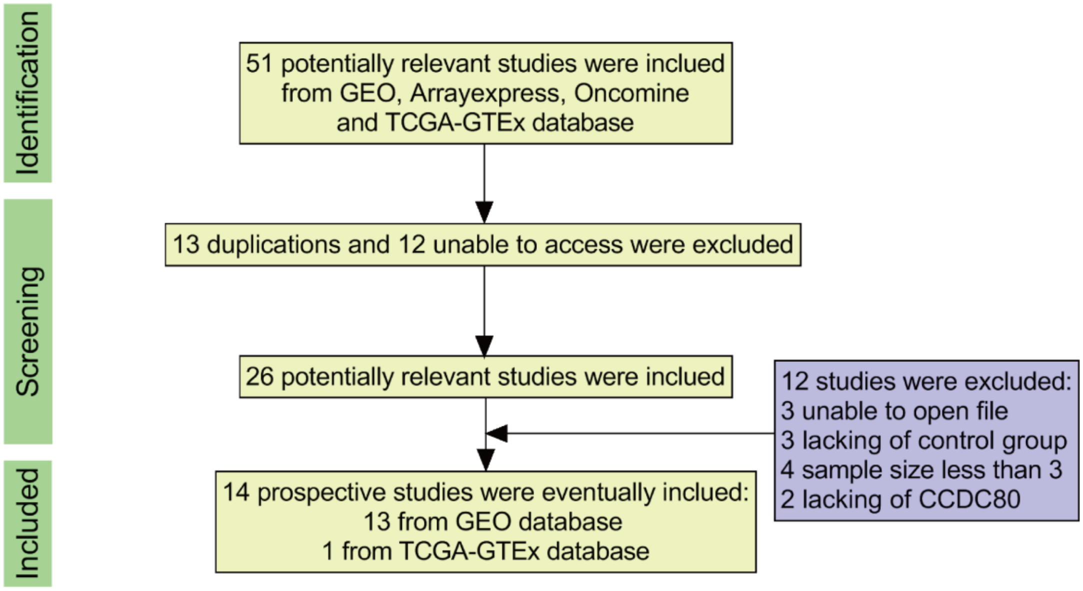

Figure S1. The flow chart of screening OVCA-related datasets for this study

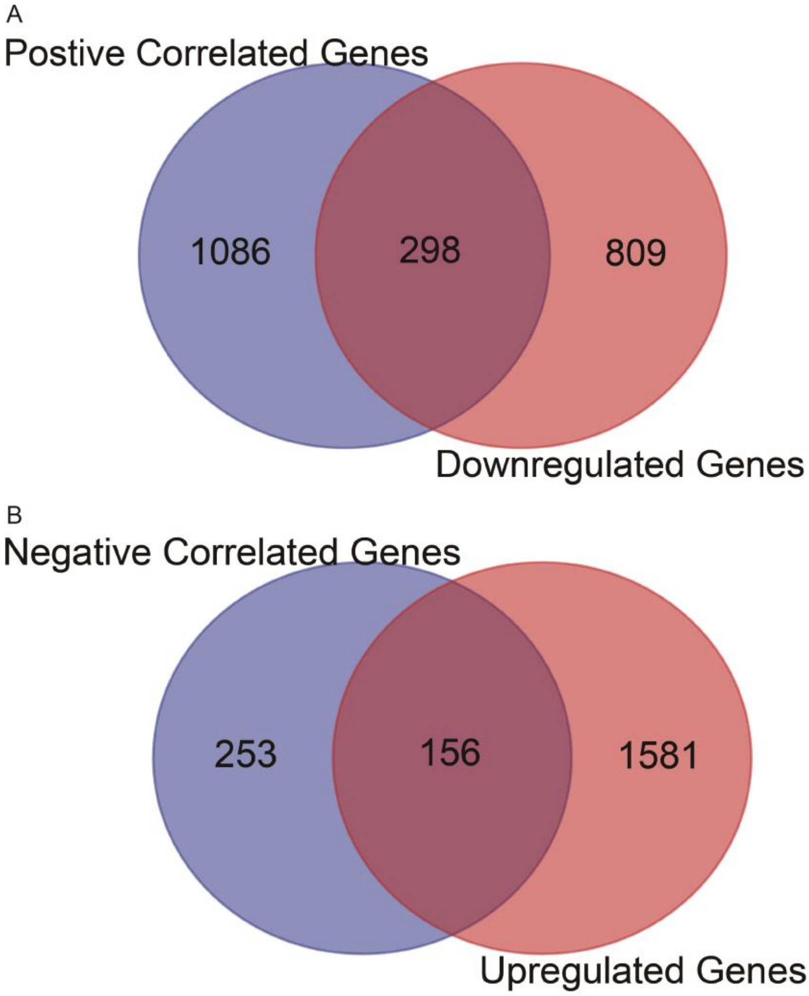

Figure S2. Venn diagrams for the intersection genes consists of *CCDC80* positively related CEGs and downregulated DEGs (A), and intersection genes consists of

CCDC80 negatively related CEGs and upregulated DEGs (B)

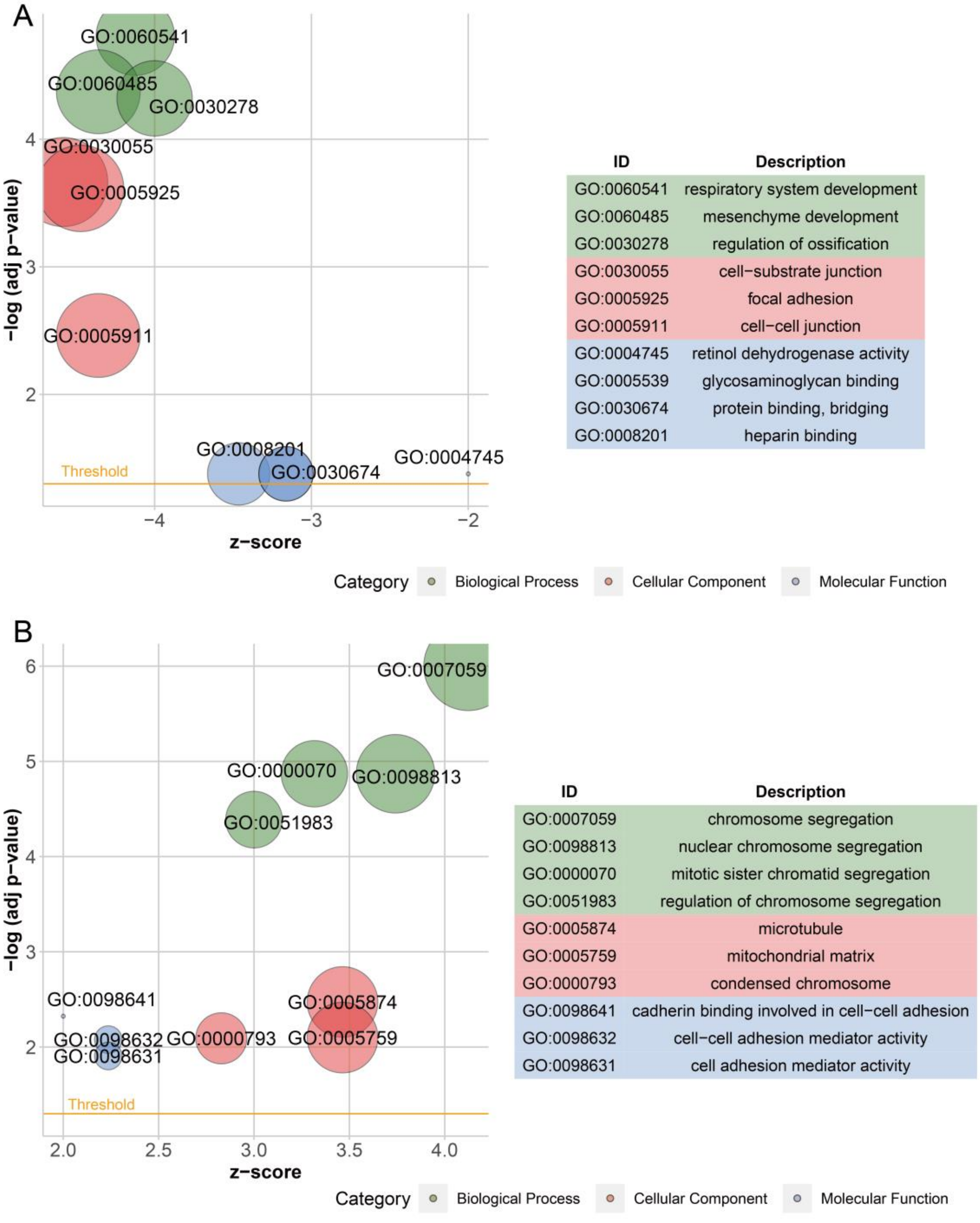

Figure S3. Bubble plots of GO annotation analysis based on intersection genes from *CCDC80* positively related CEGs and downregulated DEGs (A), and *CCDC80* negatively related CEGs and upregulated DEGs (B)

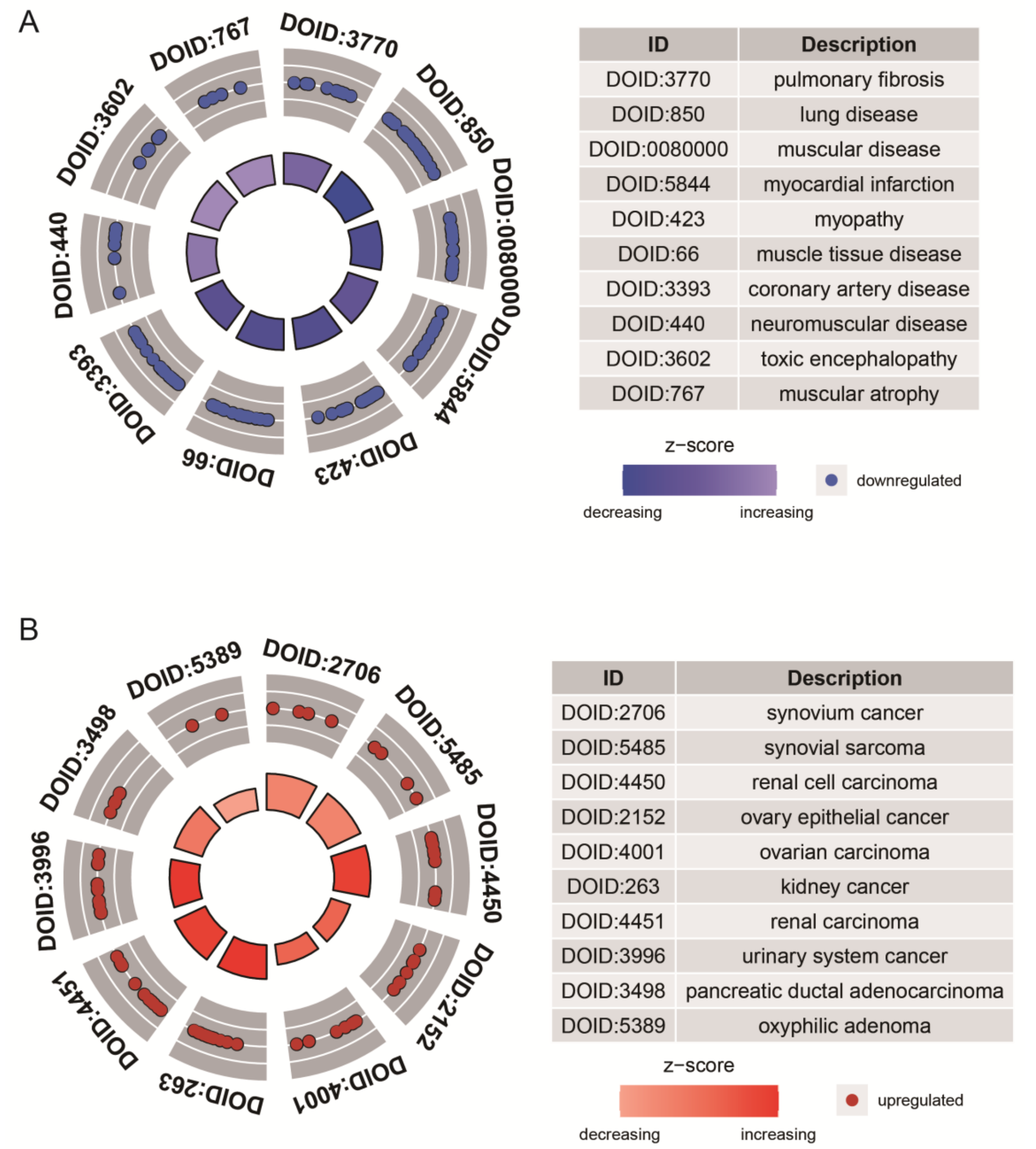

Figure S4. Circle plots of DO annotation analysis based on intersection genes from *CCDC80* positively related CEGs and downregulated DEGs (A), and *CCDC80* negatively related CEGs and upregulated DEGs (B)

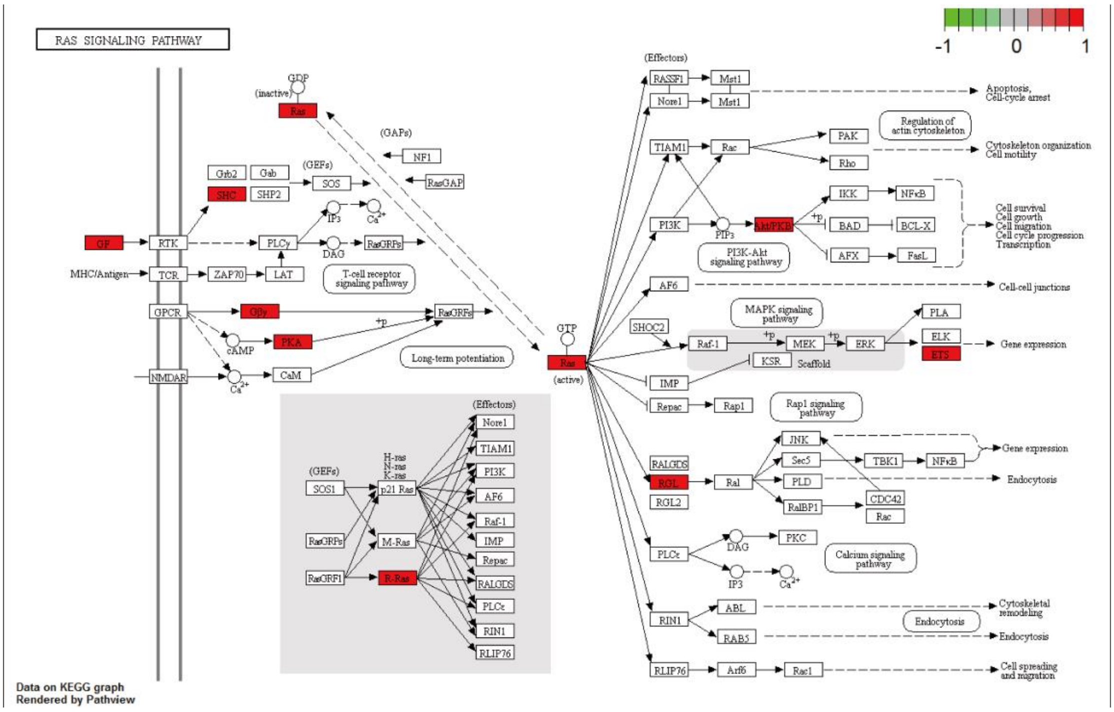

Figure S5. Visualization of Ras signaling pathway

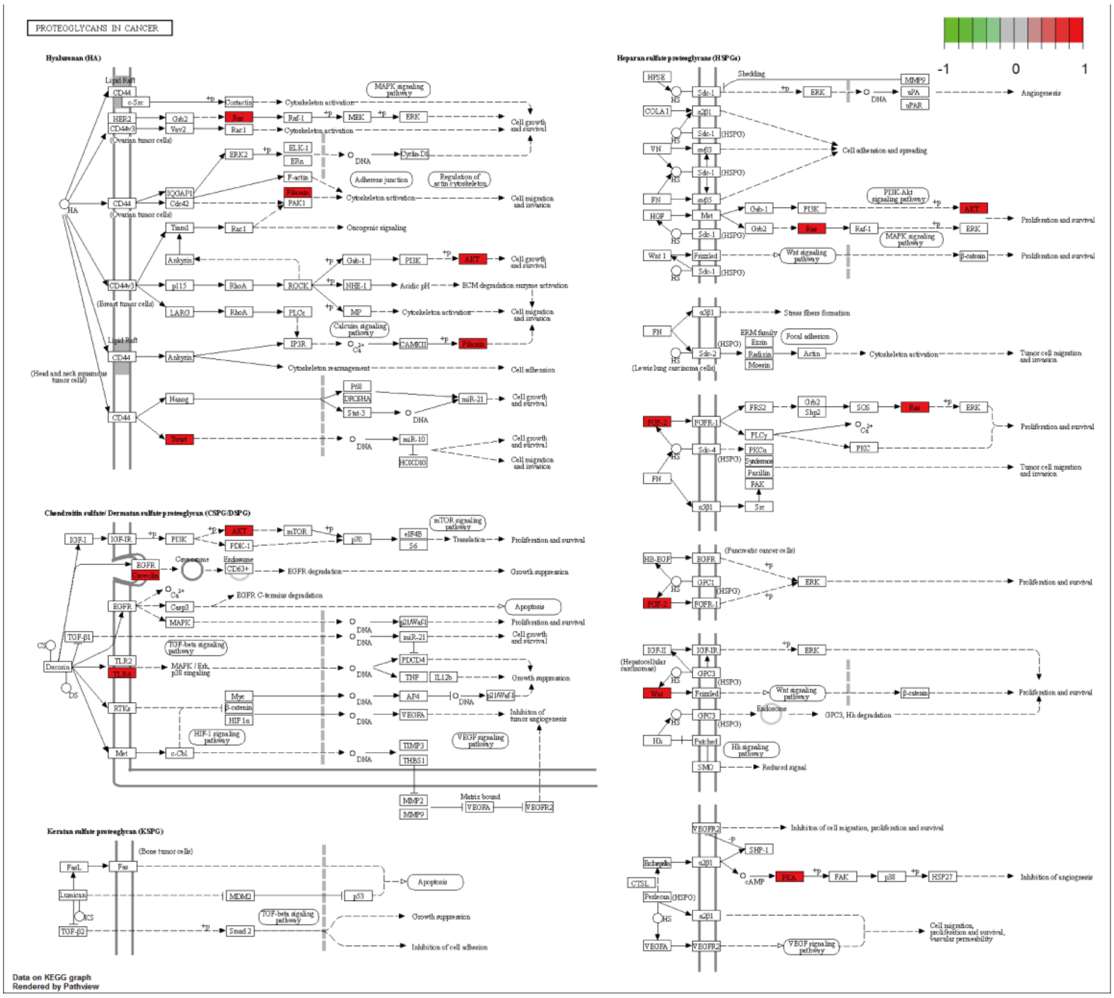

Figure S6. Visualization of proteoglycans in cancer pathway

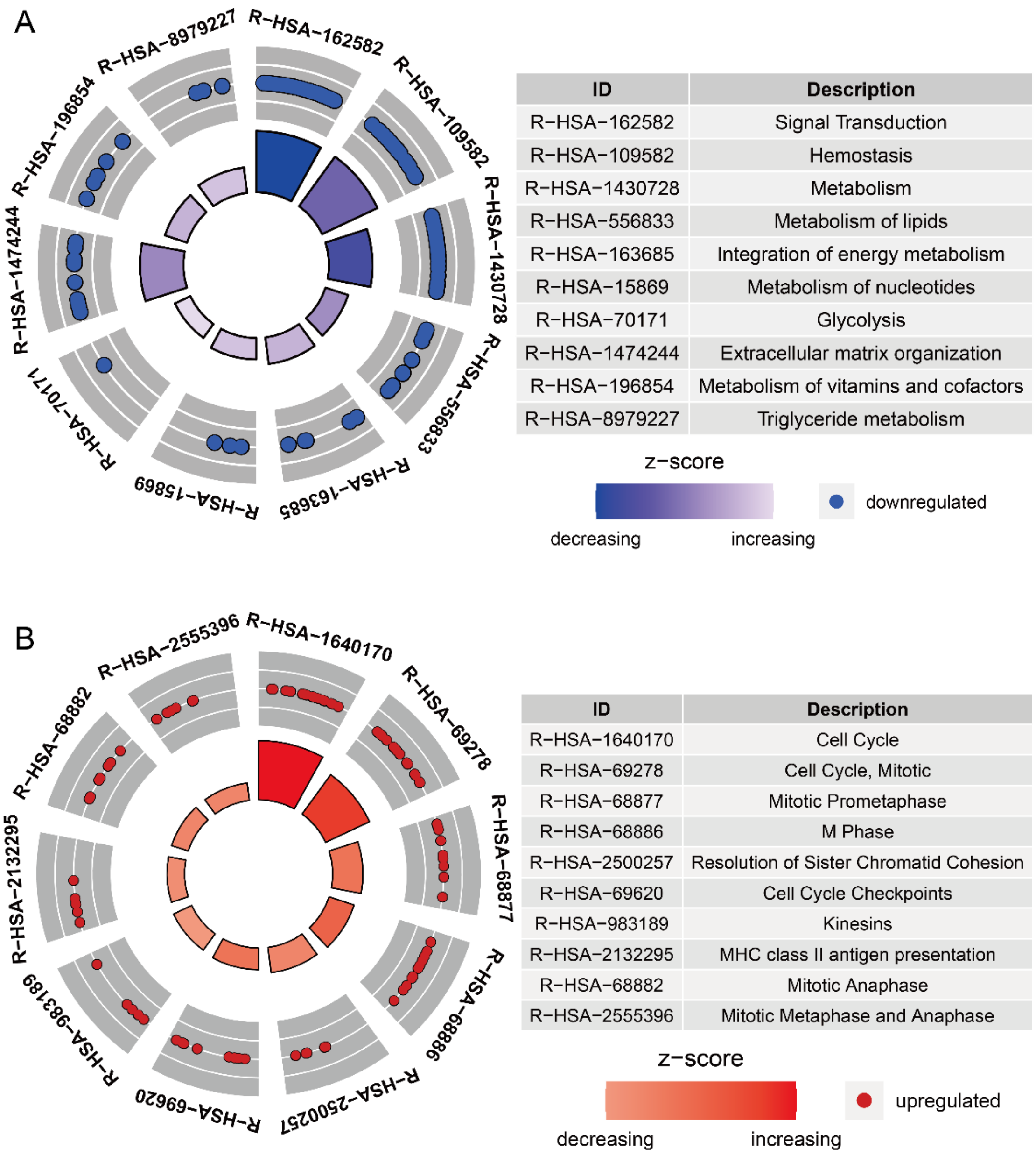

Figure S7. Circle plots of Reactome pathway analysis based on intersection genes from *CCDC80* positively related CEGs and downregulated DEGs (A), and *CCDC80* negatively related CEGs and upregulated DEGs (B)

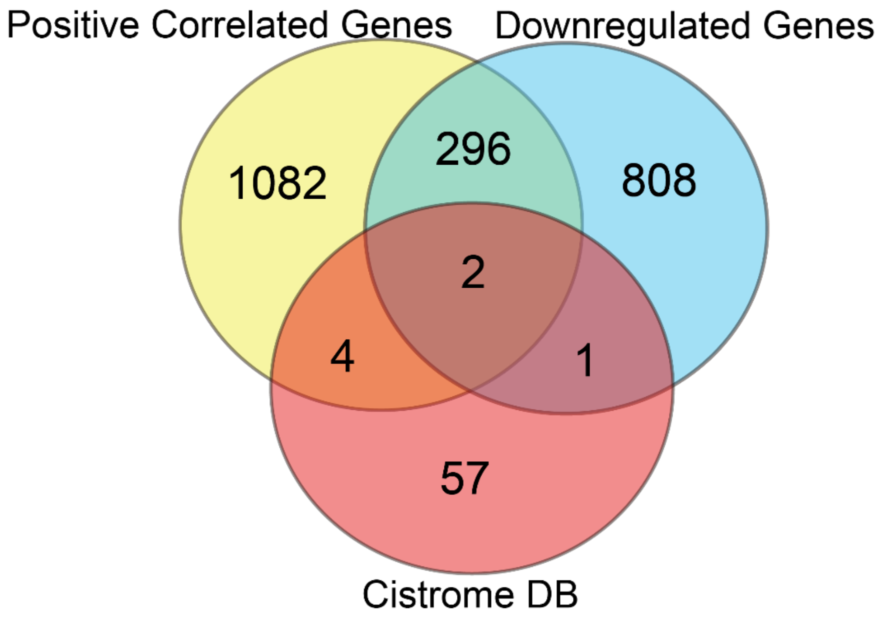

Figure S8. Venn diagrams for the intersection genes consists of *CCDC80* positively related CEGs, downregulated DEGs and predicted TFs from Cistrome DB
